# Prognostic models predicting clinical outcomes in patients diagnosed with visceral leishmaniasis: a systematic review

**DOI:** 10.1136/bmjph-2024-001196

**Published:** 2026-03-27

**Authors:** James Patrick Wilson, Forhad Chowdhury, Shermarke Hassan, Elinor Harriss, Fabiana Alves, Ahmed Musa, Prabin Dahal, Kasia Stepniewska, Philippe J Guérin

**Affiliations:** 1 Nuffield Department of Medicine, Infectious Diseases Data Observatory, University of Oxford, Oxford, UK; 2 Nuffield Department of Medicine, Centre for Tropical Medicine and Global Health, University of Oxford, Oxford, UK; 3 Bodleian Health Care Libraries, University of Oxford, Oxford, UK; 4 Drugs for Neglected Diseases initiative, Geneva, Switzerland; 5 Institute of Endemic Diseases, University of Khartoum, Khartoum, Sudan

**Keywords:** Disease Transmission, Infectious, Endemic Diseases, Epidemiologic Methods

## Abstract

**Background:**

Visceral leishmaniasis (VL) is a neglected tropical disease prevalent in populations affected by poverty and poor nutrition. Without effective treatment, death is the norm. Prognostic models can steer clinical decision-making by identifying patients at high risk of adverse outcomes. We aimed to identify, summarise and critically appraise prognostic models predicting future clinical outcomes in patients with VL.

**Methods:**

We systematically reviewed all studies that developed, evaluated or updated prognostic models predicting future clinical outcomes in patients diagnosed with VL. Five bibliographic databases (Ovid Embase, Ovid MEDLINE, Web of Science Core Collection, SciELO and LILACS) were searched from database inception to 1 March 2023, with an update to 18 December 2025. Screening, data extraction and risk of bias assessment (Prediction Model Risk of Bias Assessment Tool) were performed independently and in duplicate. Results are presented with tables, figures and a narrative synthesis.

**Results:**

Eight studies, published between 2003 and 2021, were identified describing 12 prognostic model developments. 10 models were evaluated in settings that were either geographically or temporally distinct from those used for model development, resulting in 19 external validations. All models predicted mortality, either using hospital-based cohorts (10 models) or registry data (2 models), and were developed in either Brazilian or East African populations (9 and 3 models, respectively). Model discrimination (c-statistics) ranged from 0.56 to 0.93 when evaluated in the same patients used for model development (apparent performance, 12 models), and 0.62 to 0.92 when evaluated in new settings (19 external validations, 10 models). All model developments and evaluations were judged at high risk of bias: no studies presented calibration plots, 11 models were at high risk of overfitting due to small sample sizes, and four models presented risk scores that did not correspond to the reported regression coefficients.

**Conclusions:**

All identified models predict mortality and were developed in Brazilian or East African patient populations. No prognostic models were identified that predict treatment failure or relapse, and despite South Asia accounting for the highest global VL burden prior to 2010, no models were developed in this population. Within the context of the ongoing elimination programmes in South Asia and East Africa, these represent important evidence gaps where new model development should be prioritised. Information presented in this review can be used by clinicians and policymakers to assess the applicability of existing models to their own patient settings. However, with a high risk of bias identified for all models, caution should be exercised when interpreting model risk and performance estimates. We direct interested readers to expert guidance to support transparent reporting and reduce common sources of bias in the development and evaluation of prediction models.

**PROSPERO registration number:**

CRD42023417226.

**WHAT IS ALREADY KNOWN ON THIS TOPIC**

- Visceral leishmaniasis (VL) is a neglected tropical disease associated with high mortality and predominantly affecting populations in resource-constrained settings.
- Identification of patients at high risk of adverse outcomes is critical for prioritising limited healthcare resources, including hospital admission, treatment selection and follow-up care.
- Risk stratification of patients can be performed using prognostic models; however, the range of available models and their key methodological characteristics have not been systematically evaluated.

**WHAT THIS STUDY ADDS**

- Using existing reporting guidelines for systematic reviews of prediction model studies, this review provides the first comprehensive synthesis of prognostic models predicting future clinical outcomes in patients with VL.
- We identified 12 prognostic models, all of which predict mortality and were developed in Brazil or East Africa.
- All identified models and model evaluations were judged to be at high risk of bias; therefore, model predictions and performance measures should be interpreted with caution.

**HOW THIS STUDY MIGHT AFFECT RESEARCH, PRACTICE OR POLICY**

- This review highlights key evidence gaps in the VL prediction model landscape and supports researchers in identifying candidate models for external validation or updating using local patient data.
- By summarising and appraising available models, this review enables clinicians and policymakers to assess the applicability of existing models to their own patient populations.
- By identifying common methodological limitations, this review encourages researchers to review contemporary guidance on the reporting of prediction model development and evaluation.

## Introduction

 Visceral leishmaniasis (VL) is a parasitic disease transmitted through the bite of an infected sand fly and disproportionately touches vulnerable people affected by poverty, malnutrition and forced migration.^1^ Considered a neglected tropical disease by the WHO, VL typically presents insidiously with fever, splenomegaly and weight loss and is almost universally fatal without effective treatment. The WHO estimates an annual incidence of 50 000 to 90 000 cases, although accurate estimates are obfuscated by incomplete country-level reporting.^1 2^ Despite substantial progress over the last two decades, successful treatment remains challenged by drug availability, prolonged regimens requiring hospitalisation and frequent drug side effects.^3^ Patients with previous treatment failure or immunosuppressive comorbidities, including advanced HIV infection, experience particularly high mortality and relapse rates.^1 4^

In endemic settings, accurately identifying patients at high risk of adverse outcomes is paramount when prioritising limited resources, including admission, treatment selection and follow-up intensity. Prognostic models—most commonly developed using multivariable regression techniques—estimate an individual patient’s probability of experiencing a future clinical event.^5^ Often presented as simplified risk scores, such models abound in the medical literature and inform clinical decision-making and guideline development.^6 7^ In infectious diseases alone, systematic reviews have identified over 600 prognostic models for COVID-19,^8^ 37 models for tuberculosis^9^ and 27 models for malaria.^10^ However, concerns have been raised on the methodological quality and reporting of prediction models. Biased models may overestimate performance, generate misleading predictions and ultimately contribute to suboptimal or inequitable clinical decisions.^6 7 11^

Several prognostic models have been developed and implemented for patients with VL.^12 13^ In Brazil, national guidelines introduced in 2011 recommend the use of four related risk scores, based on combinations of clinical and/or laboratory factors, to guide hospital admission and the use of liposomal amphotericin B.^13^,^16^ Similarly, since 2003, Médecins Sans Frontières (MSF) Holland has used simple VL risk scores in South Sudan to support clinical decision-making regarding liposomal amphotericin B therapy, broad-spectrum antibiotics, blood transfusions and nutritional support.^12 17 18^ Additional models exist,^19 20^ although the full range of models, including their characteristics, comparative performance and inherent biases, has yet to be systematically described.

We therefore conducted a systematic review to identify, summarise and critically appraise prognostic models predicting future clinical outcomes in patients with VL. This review aims to support policymakers in evaluating the incorporation of prognostic models into treatment guidelines, and to help clinicians assess the applicability of existing models to their own patient populations. In addition, researchers may use this review to identify evidence gaps and to determine whether available data are better suited to the development of new models, or to the external validation (evaluation) and/or updating of existing models.^21^

A glossary of key terms relating to model development and evaluation is presented in table 1.

**Table 1:** Key terms relating to prediction model development and evaluation

Term	Description
Prediction model	An equation or algorithm that estimates an individual’s probability of an outcome based on two or more predictors. Traditionally developed using multivariable regression, although machine learning methods are increasingly used.
Outcome	The event being predicted. Also termed the response or dependent variable. Models are described as prognostic when outcomes occur after the time of model use, and diagnostic when outcomes are present at the time of model use.
Predictors	Patient or group characteristics used to estimate an outcome, also termed covariates, inputs, determinants or independent variables. Predictors may be *candidate predictors* (considered during model development) or final predictors (retained in the final model).
Measures of model performance	
Overall performance	An overall summary measure of how well a model fits the data. Commonly presented measures include explained variation (R^2^) and the Brier score.
Discrimination	The ability of a model to distinguish between individuals with and without the outcome. For binary outcomes, this is often quantified using the concordance (c-)statistic, also termed the AUC, and defined as the probability that the model assigns a higher predicted risk to an individual with the outcome than to one without. Values range from 0.5 (no better than chance) to 1.0 (perfect discrimination).
Calibration	The agreement between predicted risks and observed outcomes. For binary outcomes, calibration is best assessed using a calibration plot comparing predicted risks with observed outcome frequencies across the range of predictions.
Approaches to evaluating prediction model performance	
Apparent performance	Model performance evaluated using the same dataset in which the model was developed. Performance can be optimistically biased due to overfitting, particularly in small samples or when data-driven predictor selection is used.
Internal validation	Model performance evaluated in the population represented by the development dataset, ideally using resampling techniques (eg, cross-validation or bootstrapping) to account for overfitting. Split-sample approaches are generally considered inefficient.
External validation	Model performance evaluated in new data that were not used for model development, and providing an assessment of model generalisability to new populations or settings.

Adapted from Collins *et al*^50^ and Moons *et al*.^7^

AUC, area under the curve.

## Methodology

### Protocol and registration

A protocol for this systematic review has been published^22^ and registered (PROSPERO registration number: CRD42023417226). Following peer-review feedback, we excluded the identification of systematic reviews of prognostic factor studies from the originally proposed eligibility criteria.

We adhere to Transparent Reporting of Multivariable Prediction Models for Individual Prognosis or Diagnosis: Checklist for Systematic Reviews and Meta-Analyses (TRIPOD) when reporting this systematic review.^23^ Data extraction is guided by the Checklist for Critical Appraisal and Data Extraction for Systematic Reviews of Prediction Modelling Studies (CHARMS)^24^ and the Prediction Model Risk of Bias Assessment Tool (PROBAST).^6 25^ Risk of bias assessment is performed with PROBAST.^6 25^

### Eligibility criteria

We follow a Population, Index model, Comparator model, Outcomes, Timing, Setting approach to frame our review question and define our eligibility criteria.^24 26^

The population consists of all human patients with a confirmed or suspected diagnosis of VL, as defined by the study authors. Index models include all prognostic models developed in patients with VL, including model development studies, external validation studies and/or model updating studies. No individual comparator model is defined, given that the aim of the review is to summarise and critically appraise all identified model developments and evaluations. All clinical outcomes are considered that occur following the intended time of model use, with no upper limit on the prediction horizon. The timing of model use is either at the time of, or following, VL diagnosis. We impose no restriction on the setting of model development or evaluation.

In accordance with best practice in prediction modelling research,^11 25 27^ we define a prognostic model as a multivariable model (including two or more predictors) developed with the intention of predicting future outcomes at the individual patient level. Prediction model studies are distinguished from predictor finding or prognostic factor studies, where the aim is to investigate the effect of a single or group of factors on an outcome of interest.^28^ We exclude unpublished studies (including conference abstracts, educational theses), studies that only report diagnostic prediction models and animal studies.

### Information sources and search strategy

An information specialist (EH) created the search strategy to retrieve relevant records from the following databases: Ovid Embase, Ovid MEDLINE, the Web of Science Core Collection, SciELO and LILACS. No language restrictions were imposed. The databases were initially searched from database inception to 1 March 2023. Using the same strategy, the search was updated to include additional records from 1 March 2023 to 18 December 2025. The search strategy used text words and relevant indexing terms to retrieve studies describing eligible prognostic models. The Ingui search filter was augmented with an additional search string as described by Geersing *et al*^29 30^ and combined with VL-specific keywords. Google Scholar was used to identify any complementary grey literature (full search strategy presented in online supplemental Text 1).

### Study selection

Deduplication and screening of references were performed in Covidence.^31^ Screening was performed independently by two reviewers (JPW, FC); initially at title and abstract level, and subsequently at full-text level. Where discordance existed, a third expert reviewer (PD) was consulted to make the final judgement.

Subsequent forward and backward citation searching was performed to identify records missed by the initial search.

### Data collection process

Study information was captured using a REDCap server hosted at the University of Oxford.^32^ A data extraction form was created and piloted as per the CHARMS checklist and PROBAST (online supplemental table 1).^6 24 25^ Two reviewers (JPW and SH) independently extracted the study information. Where discordance remained after discussion, a final decision was made by a third expert reviewer (PD). Study authors were not contacted in the event of unclear or missing information.

### Risk of bias

Risk of bias was assessed using PROBAST.^6 25^ Two reviewers (JPW and SH) independently assessed each model development (including updating) and external validation, by answering 20 signalling questions across four domains (participants, predictors, outcome and analysis). Responses were used to judge the overall risk of bias as either ‘low’, ‘high’ or ‘unclear’. Any discordance was resolved through discussion.

As part of the bias assessment, we established whether the model predictors and their corresponding risk scores were consistent with the reported multivariable regression coefficients (PROBAST signalling question 4.9). Consistency was assessed by referring to the study’s reported methodology, and expert guidance on the presentation of clinical risk scores.^33^

### Applicability

Applicability was not formally assessed given the broad remit of the research question. Instead, we summarise a range of information to facilitate comparison of each model’s predictors, participants and outcomes, with those of the intended target setting.^6^

### Synthesis of results

Key characteristics of the identified models and their evaluations are summarised at the aggregate study and model levels, with complementary information presented in accompanying tables and supplemental material. As all identified models predict mortality, a figure is included to facilitate comparison of candidate and final predictors across models.

A complementary narrative summary is also provided (online supplemental Text 2), where each study is described individually to provide additional contextual detail.

All identified models reported discrimination using the c-statistic. Accordingly, median and range values are presented for models evaluated in the development dataset (apparent performance) and in new patient data (external validation). Where c-statistics or other measures were not explicitly reported, no attempt was made to derive them from other performance measures. Owing to sparse and heterogeneous reporting across studies, other reported performance measures were not suitable for quantitative synthesis and are instead reported separately for individual models where available.

Meta-analysis was not performed due to substantial heterogeneity across models, including differences in predictors, populations and outcome definitions, precluding meaningful pooling of c-statistics.

### Patient and public involvement

No patients or members of the public were involved in this systematic review.

## Results

### Study and model selection

**Figure 1:**
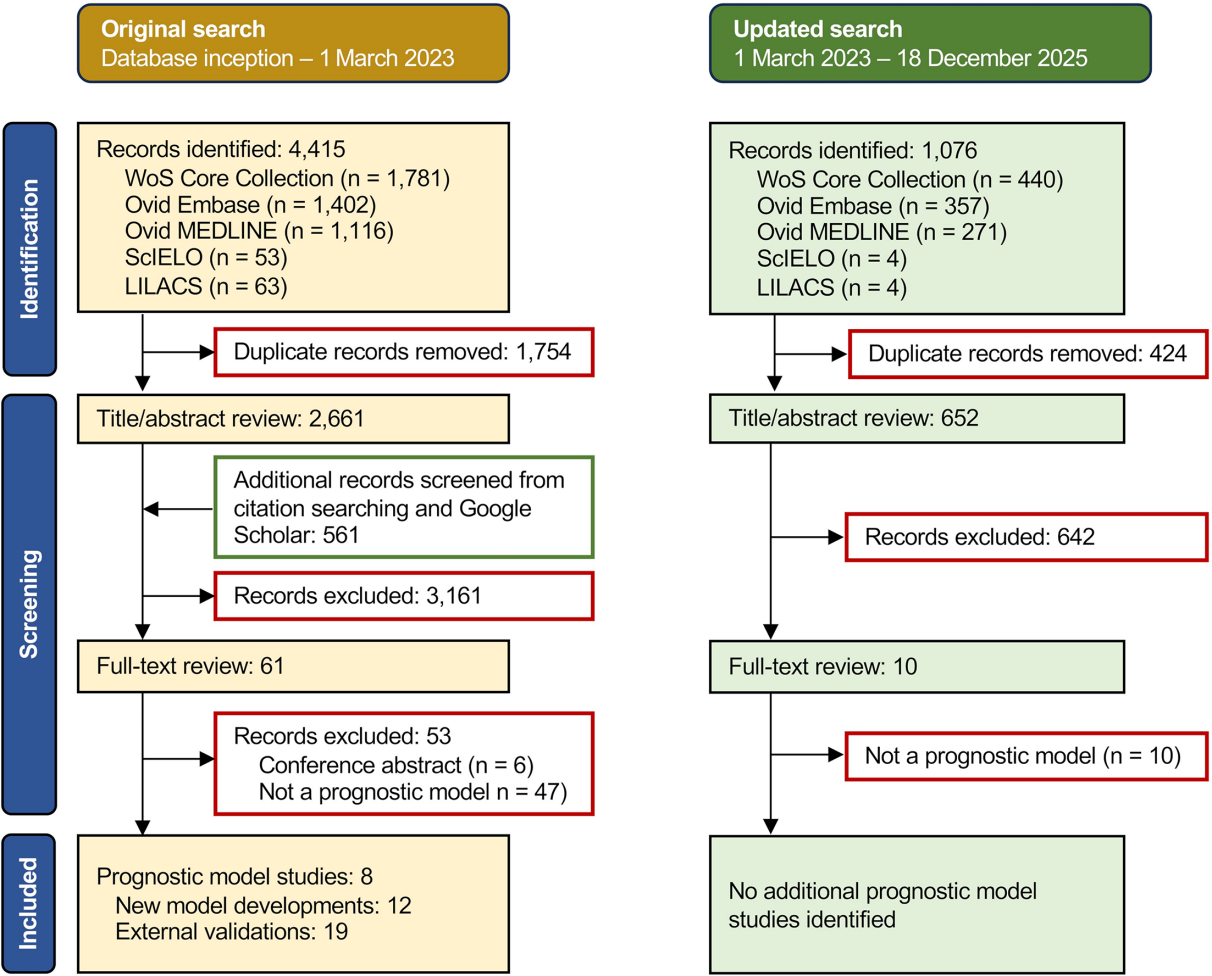
PRISMA-like flow diagram depicting the record screening process, performed initially on 1 March 2023 with subsequent updating on 18 December 2025. PRISMA, Preferred Reporting Items for Systematic Reviews and Meta-Analyses.

After deduplication, 3313 records were identified from the combined original and updated literature searches (figure 1). Title and abstract screening yielded 71 records for full-text review, of which eight prognostic model studies were identified.^1213 19 20 34^,^37^ In total, 12 prognostic models were described, of which 10 underwent one or more evaluations in patient data from either different settings and/or time periods (19 external validations presented in four studies).^12 13 36 37^

### Model developments

Key characteristics of the identified models are summarised in table 2 (with further predictor, participant and outcome information presented in online supplemental table 2). All models use multivariable logistic regression and predict mortality as a binary outcome, reported either as in-hospital mortality (10 models)^1213 20 35^,^37^ or registry-reported mortality (two models).^19 34^

**Table 2:** Key characteristics of the 12 prognostic model developments, ordered by outcome and year published

Study	Model described	Data source	Location **Period**	% male	% HIV positive	Events* Sample size (%)	Predictors†‡ Final, Candidate	EPP‡§
**Outcome: Registry-reported mortality**								
de Araújo 2012^34^	–	Registry	**Brazil (Belo Horizonte**) 2007–2009	–	–	**49** 376 (13.0%)	**4**, 48	1.0
Coura-Vital 2014^19^	–	Registry	**Brazil (Nationwide**) 2007–2011	61.7%	7.0%	**770** 12 333 (6.2%)	**12**, (29)	(26.6)
**Outcome: In-hospital mortality**								
Werneck 2003^20^	–	Case–control	**Brazil (Teresina**) - (<2003)	68.9%	–	**12** 90 (13.3%)	**4**, (15)	(0.8)
Sampaio 2010^35^	–	Retrospective	**Brazil (Recife**) 1996–2006	50.4%	–	**57** 546 (10.4%)	**6**, (15)	(3.8)
Costa 2016^13^	<2 years, clin only	Prospective	**Brazil (Teresina**) 2005–2008	–	–	**–§** 314 (¶%)	**6**, (25)	(0.9)
	<2 years, clin+lab	Prospective	**Brazil (Teresina**) 2005–2008	–	–	**–§** 291 (¶%)	**6**, (31)	(0.7)
	≥2 years, clin only	Prospective	**Brazil (Teresina**) 2005–2008	–	–	**–§** 569 (¶%)	**9**, (27)	(1.6)
	≥2 years, clin+lab	Prospective	**Brazil (Teresina**) 2005–2008	–	–	**–§** 538 (¶%)	**9**, (33)	(1.2)
Abongomera 2017^12^	–	Retrospective	**Ethiopia (Abdurafi**) 2008–2013	95.9%	19.3%	**99** 1686 (5.9%)	**8**, 16	6.2
Kämink 2017^36^	<19 years	Retrospective	**South Sudan (Lankien**) 2013–2015	54.2%	excl.	**116** 4931 (2.4%)	**8**, 20	5.8
	≥19 years	Retrospective	**South Sudan (Lankien**) 2013–2015	56.2%	excl.	**70** 1702 (4.1%)	**8,** 21	3.3
Foinquinos 2021^37^	Sampaio updating	Retrospective	**Brazil (Recife**) 2008–2018	48.7%	–	**10** 156 (6.4%)**	**1**, 1	10.0

Each row corresponds to a different model developed by the referenced study.

* Including patients with missing predictor information, excluding patients with missing/excluded outcomes.

† Number of predictor parameters (degrees of freedom).

‡ Predictor and EPP (events per predictor parameter) presented in brackets are estimated from incomplete reporting.

§ In Costa 2016, total events in combined development dataset reported as 7.5% (66/883) and not disaggregated by age group. EPP is calculated based on extrapolating the 7.5% event rate to each individual model development dataset.

¶ Not reported for development dataset.

** Sample size excludes both participants with missing predictors and missing/excluded outcomes.

clin, clinical; EPP, events per predictor parameter; excl, excluded; lab, laboratory.

Two studies (three models) were developed in East African MSF treatment centres (one model from Ethiopia that included patients with HIV/VL co-infection,^12^ and two models from South Sudan, for patients ≥19 and <19 years, and excluding HIV/VL co-infection^36^). The remaining six studies (nine models) were performed in Brazil. Two Brazilian studies (two models) were developed using registry data; either at a national level^19^ or for residents of Belo Horizonte, state of Minas Gerais.^34^ The remaining Brazilian studies were developed in hospital settings, including two studies (five models) developed from patients admitted to a hospital in Teresina,^13 20^ and two studies (two models) that were developed for children <15 years and admitted to a hospital in Recife, state of Pernambuco.^35 37^ No models were developed in South Asia or the Mediterranean region.

Most studies employed a retrospective cohort design, using hospital records (four studies, five models)^1235^,^37^ or registry data (two studies, two models).^19 34^ One study used a prospective cohort design (four models)^13^ and one study (one model) used a case–control design.^20^ The median number of patients used for model development was 542 (range 90–12 333).

Participant age formed the inclusion criteria of eight models,^1335^,^37^ with five models limiting inclusion to adolescents and younger.^1335^,^37^ Where reported, the median proportion of male participants was 56.2% (range 48.7%–95.9%, seven models). No model excluded participants based on sex. Of the models developed in adults (≥15 years), patients living with HIV were either excluded (two models),^36^ not reported (two models),^20 34^ and where reported, ranged from 7.0% to 19.3% of the model development datasets (four models).^12 13 19^

### External validations

Both East African studies performed external validations of their model developments (two studies, three models).^12 36^ The model developed in Ethiopia (Abdurafi health centre, Abdurafi, Amhara region) was validated using data from a nearby treatment centre (Leishmaniasis Research and Treatment Centre, Gondar, Gondar, Amhara region),^12^ and the two models developed in South Sudan were validated using retrospectively collected data from the same treatment centre (Lankien hospital, Jonglei state) and a treatment centre from a neighbouring state in South Sudan (Malakal hospital, Upper Nile state) (three external validations per model).^36^

Two Brazilian studies reported the external validations of eight models.^13 37^ One study, conducted in a prospective hospital cohort (Teresina, state of Piauí), both developed and evaluated four models. All four models were evaluated in patients attending the same hospital over the following five years.^13^ The same study used their prospective cohort to evaluate three further models: one developed from a historical cohort from the same hospital,^20^ one developed from a retrospective hospital cohort (Recife, state of Pernambuco),^35^ and one using national registry data.^19^ The second Brazilian study^37^ used a retrospective hospital cohort (Recife, state of Pernambuco) to both evaluate and update a model previously developed in the same hospital.^35^

Further details on validation datasets are presented in online supplemental table 2.

### Model performance

Model discrimination measures were reported as c-statistics for all risk scores. Further performance measures, where reported, are detailed in online supplemental table 3. For external validations, the median c-statistic was 0.78 (range 0.62–0.92, 10 models, 19 external validations). When evaluated in the same patients used for model development (apparent performance), the median c-statistic was 0.86 (range 0.56–0.93, 12 models). No studies presented overall measures of performance or calibration plots. One model’s calibration plot could be reproduced from an internal (split-sample) validation of the risk score.^19^

### Predictors

**Figure 2:**
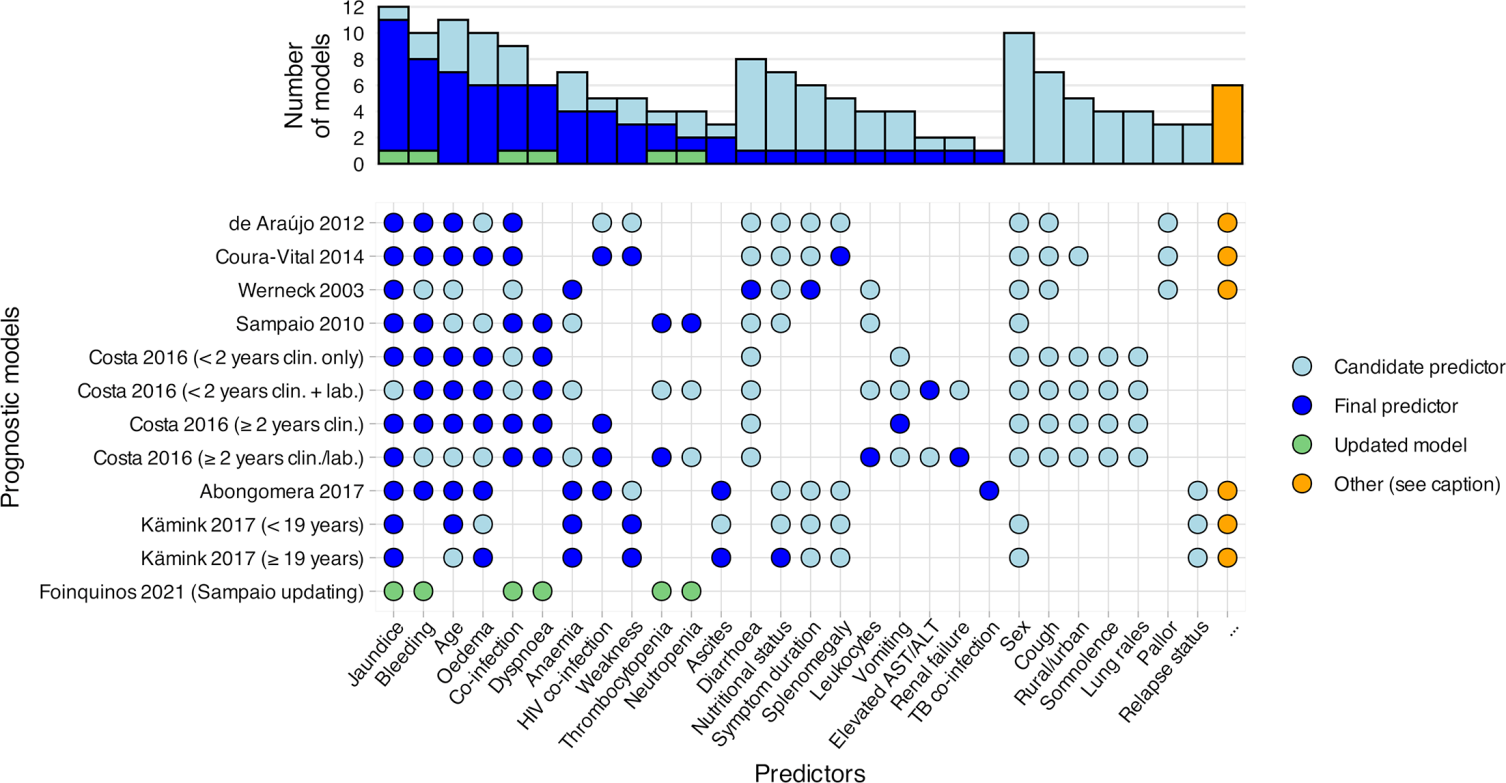
Candidate (considered) and final (retained) predictors included in prognostic models of mortality in visceral leishmaniasis. Bars indicate the number of models incorporating each predictor. Models are labelled by first author, publication year and model name (in brackets where multiple models were reported). Conceptually similar predictors were grouped and renamed; definitions and groupings as reported by the study authors are provided in online supplemental table 4. For models by de Araújo *et al*^34^ and Coura-Vital *et al*,^19^ cough and/or diarrhoea were treated as a single predictor in accordance with national registry reporting. For models by Kämink *et al*,^36^ oedema and ascites were combined as a single predictor, whereas Abongomera *et al* treated these as separate variables. Predictors shown for the updated model correspond to the predetermined final predictors of the model being updated (Sampaio *et al*).^35^ ‘…’ predictors assessed in ≤ 2 models and not included in the final model: Werneck *et al*^20^: ‘abdominal distension’, episodes of blood transfusion; de Araújo *et al*^34^: fever, hepatomegaly, ‘other clinical manifestations’, initial VL regimen, VL drugs following initial regimen, antimony treatment duration; Coura-Vital *et al*^19^: fever, hepatomegaly, ‘other clinical manifestations’, race, education; Abongomera *et al*^12^: initial VL regimen; Kämink *et al*^36^ both models]: lymphadenopathy. ALT, alanine transaminase; AST, aspartate transaminase; clin., clinical; lab., laboratory; TB, tuberculosis.

A visual comparison of the candidate and final predictors is presented in figure 2. Where authors provide further predictors’ definitions, these are reported in online supplemental table 4. The four most common candidate predictors were jaundice (12 models), age (11 models), sex (10 models) and bleeding (10 models). Initial VL treatment was included as a candidate predictor in two models, although not retained in the final models.^12 34^ Predictors most frequently retained in the models were jaundice (11 models), bleeding (8 models) and age (7 models). No models included sex as a final predictor. One model did not include HIV status as a candidate predictor, despite being conducted in adults and not prespecifying the exclusion of patients with HIV.^20^ Apart from HIV testing, four models did not consider laboratory tests as a predictor.^13 19 34^ The remaining eight models included laboratory tests both as candidate and final predictors.^1213 20 35^,^37^

### Model presentation

All 12 models were presented as simplified risk scores. Online supplemental table 5 describes the score ranges, suggested risk groupings and the authors’ suggestions on how different risk groups should inform clinical decision-making. Outcome (mortality) probabilities corresponding to the risk scores were presented either in tabular format (four models),^12 19 36^ graphically and through a web application (four models),^13^ or not presented (four models).^20 34 35 37^ The full model equation, including model intercept, was reproducible for three models in total—presented either in the original study describing the model development (one model),^19^ or in the updating study (two models, corresponding to the original model and updated model).^37^

### Risk of bias assessment

Risk of bias assessments for all model developments and external validations are presented in table 3, alongside measures of discrimination and details of model presentation and reproducibility. Online supplemental Table 3 presents further details on variable selection and handling of missing data. Responses to the risk of bias signalling questions are provided in online supplemental table 6.^38^

**Table 3:** Summary of model discrimination and risk of bias assessments, ordered by outcome and year published

Study	Model described	Model discrimination (c-statistic)*			Risk of bias†		
		Apparent performance	Internal validation	External validation	Eval. type	P/Pr/O/A	OA
**Outcome: registry-reported mortality**							
de Araújo 2012^34^	–	0.756	–	–	dev	+/+/?/+	+
Coura-Vital 2014^19^	–	0.80 (0.78–0.82)	0.78 (0.75–0.82)‡	–	dev	+/+/?/+	+
**Outcome: in-hospital mortality**							
Werneck 2003^20^	–	0.882	–	–	dev	+/+/−/+	+
Sampaio 2010^35^	–	0.895	–	–	dev	+/+/−/+	+
Costa 2016^13^	< 2 years, clin only	0.90 (0.84–0.97)	–	0.83 (0.64–1) 0.86 (0.74–0.98)	dev val (×2)	−/−/−/+ −/−/−/+	+
	< 2 years, clin+lab	0.93 (0.88–0.98)	–	0.80 (0.57–1) 0.92 (0.84–1)	dev val (×2)	−/−/−/+ −/−/−/+	+
	≥ 2 years, clin only	0.89 (0.84–0.93)	–	0.75 (0.68–0.83) 0.88 (0.83–0.93)	dev val (×2)	−/−/−/+ −/−/−/+	+
	≥ 2 years, clin+lab	0.92 (0.88–0.96)	–	0.79 (0.62–0.96) 0.71 (0.34–1)	dev val (×2)	−/−/−/+ −/−/−/+	+
	Werneck 2003	n/a	n/a	0.75	val	?/−/−/+	+
	Sampaio 2010	n/a	n/a	0.87	val	?/−/−/+	+
	Coura-Vital 2014	n/a	n/a	0.77	val	?/−/−/+	+
Abongomera 2017^12^	–	0.83 (0.79–0.87)	0.82 (0.77–0.88)§	0.78 (0.72–0.83)	dev val (×1)	+/−/−/+ +/−/−/+	+
Kämink 2017^36^	<19 years	0.83 (0.78–0.87)	–	0.72; 0.83; 0.77	dev val (×3)	+/+/−/+ +/+/?/+	+
	≥19 years	0.74 (0.68–0.81)	–	0.72; 0.80; 0.71	dev val (×3)	+/+/−/+ +/+/?/+	+
Foinquinos 2021^37^	Sampaio updating	0.556 0.762 (0.662–0.901)¶	–	–	dev	+/+/−/+	+
	Sampaio 2010	n/a	n/a	0.618¶	val (×1)	+/+/−/+	+

Each row corresponds to a different model that was either developed or externally validated by the referenced study.

* 95% CIs are reproduced as reported.

† Where a study presents multiple external validations of the same model, risk of bias assessments were the same and therefore grouped together.

‡ Split-sample (random, 2:1 development:validation).

§ Cross-validation (fivefold).

¶ Assessing performance of the full model equation.

?, unclear risk of bias; −, low risk of bias; +, high risk of bias; c-statistic, concordance-statistic; dev, development; N, no; n/a, not applicable; O, outcome; OA, overall assessment; P, participants; Pr, predictors; val, validation.

All 12 model developments were judged at an overall high risk of bias.

The analysis domain was assessed at high risk of bias across all model developments. One model obtained a sufficient sample size (event to predictor parameter ratio > 10), and adequately reported model performance, including calibration.^19^ All models (excluding model updating) were developed using a univariable selection stage and did not adjust model predictions to account for optimism due to overfitting. In four model developments, the presented risk scores were not reproducible from the regression model coefficients^13^ (full calculations reported in online supplemental Text 3, further elaboration in online supplemental Text 2).

Five models (two studies) were assessed as having a low risk of bias in the predictors domain.^12 13^ Both studies provided evidence that the model predictors were defined consistently for all patients and were assessed without knowledge of outcome data. The remaining seven models (six studies) were considered at high risk of bias, with one model including predictors that were likely measured after the time of intended model implementation.^34^

The outcome domain was assessed at low risk of bias for all model developments, except for two models where bias risk was unclear.^19 34^

The participants’ domain was assessed at low risk of bias for four models (one study),^13^ with the remaining models considered high risk due to using retrospectively collected data.

All 19 external validations were also judged at high risk of bias, although assessment across the domains was limited by a lack of reporting. Briefly, sources of bias were similar to those identified for model developments, including small sample sizes, the use of retrospectively collected data and the absence of calibration measure reporting. Please refer to online supplemental table 6 for further details.

## Discussion

Across a range of diseases, the number of prediction model studies has surged over the last two decades, driven by an increasing focus on personalised medicine, the need to provide evidence for guideline development, and a growing number of tools available for model development.^9 27 39 40^ VL is no exception, with a total of 12 prognostic models identified, of which nine were published since 2013. All models were developed in Brazil or East Africa and predict either in-hospital or registry-reported mortality.

When using a prognostic model to predict mortality, for example, in a hospital ward or outpatient clinic, the clinician should be confident that after inputting model predictors (eg, age, haemoglobin, clinical signs and symptoms), they receive a trusted estimate of the probability (risk) of the outcome occurring. Empowered with this information, the patient can then be counselled, and important treatment decisions agreed on. Having confidence in the model output is fundamental, since inaccurate risk predictions can lead to suboptimal decision-making, inequitable care and, at times, patient harm.

### Prognostic model assessment

Three important considerations should be taken into account when assessing whether a prognostic model’s estimated risks are reliable for a target patient. These include (1) applicability: whether the model has been developed or evaluated in a setting and population similar to that of the target patient; (2) performance, including both discrimination and calibration, in the populations and settings chosen for evaluation and (3) risk of bias: whether the studies performing model development or evaluation are subject to systematic errors that may distort risk estimates and performance measures.

### Applicability

First, we consider model applicability.^6^ Has the model been developed and/or evaluated in patients similar to the patient I’m interested in? Here, the onus is on the model user to compare their target patient and setting to those in which the model was developed and/or evaluated (table 2, online supplemental table 2). In VL, important mismatches can result from differences in (1) HIV status, (2) patient age, (3) geographical setting (both locally within a country and between endemic regions), (4) treatments used (where reported), (5) temporal differences and (6) treatment setting (eg, inpatient vs outpatient). For example, estimating the mortality risk of a patient with HIV co-infection using a model developed from HIV-negative patients is likely to underestimate the true risk. Similarly, using a model developed in Brazil to estimate mortality risk in patients from India may well overestimate the true risk, given the overall lower mortality rate in South Asia.^1^ One may also question the contemporary appplication of risk estimates from models developed using data from 10 to 20 years ago. For example, is it fair to assume that models developed using hospital or registry data in Brazil in the 2000s and early 2010s are still accurate, given significant changes in treatment, and an evolving disease epidemiology?^41^ Questions regarding model applicability, such as these, often have no clear consensus answer, although are important to consider.

### Performance

Second, even if a model were applicable to my patient population, how can I be sure it performs well? As we summarise here (table 3 and online supplemental table 3), model discrimination, presented as the c-statistic, is universally reported across all identified models, both when evaluated in the same patients used for model development (apparent performance) and when evaluated in new patients (external validation). Given the reported c-statistics are frequently over 70%–80%, even when evaluated in new data, we are reassured that in the appropriate population, most models do a fairly good job at assigning higher risk to those who progress to death compared with those who do not. Crucially, while a highly discriminative model can reliably rank patients by risk, discrimination alone provides no information on the accuracy of the predicted probabilities. A model that estimates a mortality risk of 20%, where the true risk is 2%, may still have 100% discriminative performance, despite dramatically overestimating the true risk.

Instead, we should be reviewing a model’s calibration, ideally presented as a plot of observed vs estimated risks, to assess this fundamental, yet frequently overlooked aspect of model performance.^42^ However, with estimated and observed risks only reproducible for one model risk score,^19^ we find that the VL prognostic model landscape bucks the broader trend: calibration is neglected.^8 21 43^ In contrast, measures of discrimination and classification (sensitivity, specificity) are preferentially reported, despite their limited clinical utility.^6^

### Risk of bias assessment

Finally, we consider risk of bias, alongside the closely related issue of model reporting, both of which directly influence the interpretability, reproducibility and clinical application of prognostic models.

All model development and external validation studies were judged to be at high risk of bias according to PROBAST. Importantly, this does not imply that the identified models are inherently flawed or clinically uninformative. Rather, PROBAST highlights aspects of study design, analysis or reporting that can challenge interpretation of estimated risks and performance measures.^6^ Below, we briefly describe several recurrent issues and direct readers to accessible guidance on best practice in prognostic modelling.

A frequent concern was model overfitting, which occurs when models capture random variation specific to the development dataset rather than true underlying risk patterns. This is more likely when many predictors are considered relative to the number of outcome events and can lead to exaggerated performance estimates and inflated risk predictions when models are applied to new patients.^44 45^ While rules of thumb often suggest a minimum of 10–20 events per predictor parameter, most identified models fell well below this threshold. Formal sample size calculations are now available for prediction model development, which can also be used to assess the number of predictors that can be reliably supported for a given sample size.^46^

Internal validation provides a means of adjusting performance and risk estimates for the effects of overfitting. However, only two models applied internal validation methods, either using cross-validation^12^ or a split-sample approach.^19^ Although historically common, data splitting is increasingly discouraged, particularly in smaller datasets, as it reduces the effective sample size available for model development. Resampling methods, such as bootstrapping, allow full use of the available data while providing more reliable estimates of model performance.^7^

Another recurring source of bias was predictor selection based on univariable analyses. This approach can result in unstable models and misleading predictor inclusion, as variables are selected based on isolated statistical associations rather than their joint contribution to risk prediction. Alternative strategies include pre-specifying predictors based on clinical relevance, or applying penalisation or dimension-reduction techniques to limit model complexity.^6 47^

We also identified reporting-related concerns that directly affected reproducibility of derived risk scores, with one study (four models) presenting scores that did not correspond to reported regression coefficients. While these discrepancies may reflect reporting errors, they undermine confidence in score implementation and external use (online supplemental Text 3).

Other concerns related to model reporting and reproducibility were commonplace, limiting both model appraisal and application to the individual patient. Notably, several studies did not report absolute risk estimates corresponding to the presented risk scores.^20 34 35 37^ When models are presented primarily as binary classifiers (eg, high vs low risk), much of their potential clinical utility is lost, as clinicians are unable to interpret or communicate individualised risk estimates.

Similarly, incomplete presentation of the full model equation—including the intercept term—restricted reproducibility and external evaluation. Without this information, users are limited to applying simplified risk scores, which may differ in performance from the underlying regression model.^6^ As only two studies reported the full model equation,^19 37^ independent evaluation of the remaining models would require contacting the study authors.

Additional sources of bias—such as categorisation of continuous predictors, uncertain or suboptimal handling of missing data, reliance on retrospective data sources and model evaluation in datasets with few outcomes—were also common. Importantly, the issues identified in this review are not unique to VL, but mirror challenges repeatedly highlighted in systematic reviews of prognostic models across a wide range of disease areas.^6 8 40 48 49^ For readers interested in addressing these issues, we signpost accessible and authoritative guidance on best practices in model development,^47^ evaluation,^50 51^ sample size calculation,^46 52^ model reporting,^40^ model presentation, (including risk score derivation),^33^ and recently updated guidance on risk of bias assessment.^53^

### Predictors of mortality

Where outcome timing was reported, death frequently occurred within days of hospitalisation, indicating a short prediction horizon. Two of the included prediction model studies reported time to death: one-third of patients died within 48 hours of admission in South Sudan,^36^ and the average time to death was just over 5 days in a study reporting from Teresina (Piauí, Brazil). In this context, predictors retained across models predominantly reflect advanced disease and imminent physiological decompensation.

Severe VL is increasingly understood as a progressive inflammatory syndrome—described as ‘leishmanial sepsis’ and characterised by cytokine storm, disseminated intravascular coagulation, secondary bacterial sepsis and evolving multiple organ dysfunction.^54^ It is therefore unsurprising that frequently identified predictors of mortality—including jaundice, bleeding, dyspnoea, oedema and bacterial co-infection (figure 2)—represent clinical markers of established organ failure and broadly align with factors identified in systematic reviews of prognostic factors for VL mortality in East Africa^55^ and Brazil.^56^

Conversely, factors plausibly important earlier in the disease course, such as symptom duration, nutritional status or relapse history, were infrequently selected. While these variables may influence susceptibility or delayed care-seeking, they appear to have limited discriminatory value for mortality once accounting for disease severity at the time of initiating treatment.

The short prediction horizon also explains the relatively high discrimination reported across studies, with c-statistics frequently approaching or exceeding 0.85. Under these conditions, discrimination is driven by late-stage clinical features rather than early prognostic signals. As a result, existing models primarily identify patients at high risk of near-term mortality, rather than supporting earlier risk stratification or anticipation of clinical deterioration.

### Implications for future research

Several important evidence gaps were identified in this review. Most notably, no prediction model studies were identified outside East Africa and Brazil. This is striking given that South Asia has, until recently, accounted for the lion’s share of the global VL burden. Mortality rates in South Asia are, however, relatively low compared with Brazil and East Africa, meaning that relatively large sample sizes would be required to develop mortality models without substantial risk of overfitting. In this context, routinely collected programme data and national surveillance systems may offer a pragmatic opportunity to support adequately powered model development, provided data quality and outcome ascertainment are sufficient.

A further major evidence gap is the absence of models predicting relapse or post-kala-azar dermal leishmaniasis (PKDL). These outcomes are of particular importance in elimination settings, since both represent infection reservoirs that sustain transmission.^57 58^ The lack of current prognostic tools in this area limits our ability to target follow-up, secondary prophylaxis or intensified surveillance. Recently, the WHO released a target product profile for a test of cure following treatment, such that patients at high risk of subsequent relapse can be identified early.^59^ A prognostic model for relapse could serve as a surrogate for such an in vitro test. Current efforts are underway to develop and validate prognostic models for VL relapse using individual patient data (IPD) from clinical efficacy trials, coordinated through international data-sharing initiatives.^60^ Leveraging IPD meta-analysis allows harmonisation of data across heterogeneous studies, increases statistical power and maximises the value of existing datasets—particularly in disease areas where high-quality data are scarce.

In Brazil, most existing models—including those informing current national guidelines^16^ —are based on data collected 10–20 years ago. Since that time, the epidemiology of VL has evolved, and treatment practices have shifted substantially, for example, with higher rates of HIV/VL co-infection, an increasing age at diagnosis and expanding access to liposomal amphotericin B.^41^ These changes raise questions about the continued validity of older models and highlight the need for model evaluation with recent patient data and, where necessary, model updating. In addition, future studies should carefully consider whether it is appropriate to combine patients with and without HIV within a single model, given their distinct clinical trajectories, immunological profiles and risk factors for adverse outcomes.^61^

Finally, emerging applications of artificial intelligence (AI) and machine learning warrant cautious consideration in the VL prediction modelling landscape. Recent studies have demonstrated the feasibility of applying machine learning methods to VL datasets.^38 62^ However, as emphasised in PROBAST+AI and TRIPOD+AI guidance, such approaches are not immune to bias, overfitting or poor transparency.^40 53^ Without rigorous reporting, external validation and explicit consideration of clinical use cases, machine learning models risk offering limited real-world utility. Future research should prioritise methodological robustness, interpretability and clinical relevance over algorithmic complexity.

### Limitations

The principal limitations of this review relate to its scope. We excluded unpublished manuscripts, including conference abstracts and educational theses, due to concerns about variable methodological quality and access; however, this may have led to the omission of emerging or ongoing work.

In line with our prespecified inclusion criteria, we also excluded studies reporting diagnostic models,^63 64^ models that were not developed using a multivariable approach,^65^,^67^ prognostic factor studies^1868^,^70^ and registered but unpublished prognostic model studies (eg, NCT05602610). While these exclusions were necessary to maintain a focused research question, they limit our ability to provide a comprehensive overview of the full VL prediction research landscape.

## Conclusions

We present the first systematic review that identifies, summarises and critically appraises prognostic models for VL. Using established methodological guidance, we provide a comprehensive, objective and transparent synthesis of model characteristics, performance, applicability and risk of bias. Our findings highlight substantial gaps in the current evidence base. All identified models predict mortality, were developed exclusively in Brazil or East Africa and are almost all based on data collected over a decade ago. No models were developed in South Asia or the Mediterranean region, nor addressed relapse or PKDL—outcomes that are central to elimination efforts.

Clinicians, researchers and policymakers can refer to this review to assess the strengths and limitations of existing VL prognostic models in this highly neglected and often fatal disease. We direct interested readers to expert guidance to support transparent reporting and reduce common sources of bias in the development and evaluation of prediction models.^3340 46 47 50^,^53^

## Supplementary material

10.1136/bmjph-2024-001196 online supplemental table 1

10.1136/bmjph-2024-001196 online supplemental table 2

10.1136/bmjph-2024-001196 online supplemental table 3

10.1136/bmjph-2024-001196 online supplemental table 4

10.1136/bmjph-2024-001196 online supplemental table 5

10.1136/bmjph-2024-001196 online supplemental table 6

10.1136/bmjph-2024-001196 online supplemental file 1

10.1136/bmjph-2024-001196 online supplemental file 2

10.1136/bmjph-2024-001196 online supplemental file 3

## Data Availability

All data relevant to the study are included in the article or uploaded as supplementary information.
