# Supplementary material for: Prognostic models predicting clinical outcomes in patients diagnosed with visceral leishmaniasis: a systematic review
Source: BMJ Public Health. 2026 Mar 27;4(1):e001196. doi: 10.1136/bmjph-2024-001196 (PMC13034366; doi:10.1136/bmjph-2024-001196)
Supplement: online supplemental file 2 [file bmjph-4-1-s008.docx]

# Narrative summary of VL prognostic models

Table of Contents

[Narrative summary of VL prognostic models 1](#_Toc217385077)

[East African models 3](#_Toc217385078)

[Abongomera et al. (2017) (3) 3](#_Toc217385079)

[Kämink et al. (2017) (4) 4](#_Toc217385080)

[Brazilian models 4](#_Toc217385081)

[Costa et al. (2016) (5) 4](#_Toc217385082)

[Werneck et al. (2003) (8) 6](#_Toc217385083)

[Coura-Vital et al. (2014) (10) 6](#_Toc217385084)

[de Araújo et al. (2014) (11) 7](#_Toc217385085)

[Sampaio et al. (2010) (9) 8](#_Toc217385086)

[Foinquinos et al. (2021) (12) 8](#_Toc217385087)

[References 9](#_Toc217385088)

The aim of this narrative summary is to give readers study and model-specific information that aids contextual interpretation and assessment of each model’s *applicability* and *generalisability* to new settings. Unlike the main results section, which synthesises findings across studies, this narrative considers each study individually.

We summarise key prognostic model characteristics — study populations, candidate and final predictors, outcome definitions, and performance measures — and highlight study-specific issues relating to risk of bias and applicability where relevant.

**A few notes**

- The narrative here is not intended to be exhaustive in detail. Further information can be found in the accompanying tables and figure:
  - Table 2: Summary of key study and model characteristics.
  - Table 3: Summary of key reproducibility and performance measures, risk of bias assessments.
  - Figure 2: Visual comparison of candidate and final predictors.
  - Supplementary Table 2: Further details on the predictors, participants and outcomes across both development and validation datasets (where reported).
  - Supplementary Table 3: Further details on model-specific performance measures.
  - Supplementary Table 4: Risk score thresholds and interpretations, as suggested by the study authors.
  - Supplementary Table 5: Full risk of bias assessments with explanatory text.
  - Supplementary Table 6: Descriptions of predictors, including groupings and definitions, where provided.
  - Supplementary Text 3: Review of concordance between risk scores and regression coefficients.
- The following common sources of bias were identified across multiple models; where relevant I list the corresponding PROBAST signalling question and note exceptions in the narrative. Further explanation is available in the PROBAST elaboration and Supplementary Table 5:
  - **Signalling question 1.1**: Retrospective study designs for most cohorts.
  - **Signalling question 2.1**: Limited evidence that predictor measurements were consistently defined or measured across participants.
  - **Signalling question 4.1**: Low number of outcome events relative to the number of candidate predictors (low events per predictor parameter).
  - **Signalling question 4.2**: Inappropriate handling or categorisation of continuous predictors.
  - **Signalling question 4.5**: Predictor selection based on univariable p-values.
  - **Signalling question 4.7**: No calibration plots or insufficient calibration information presented.
  - **Signalling question 4.8**: Overfitting and optimistic performance not adequately addressed (lack of shrinkage, penalisation, or internal validation in some studies).
  - **Additional**: Several studies did not report the full regression equation, requiring contact with study authors for formal external validation.
- Measures of discrimination (c-statistics) should be interpreted cautiously because they reflect both the model and the population in which the model is evaluated. Discrimination is affected by the distribution of predictors and outcome prevalence in the evaluation dataset (spectrum effect). Consistent with methodological guidance, we avoid labelling arbitrary cut-offs (e.g. calling 0.7–0.8 “good”) and instead simply report the c-statistics with confidence intervals (2).
- All identified studies share at least one co-author with one other study, reflecting shared settings/organisations. For continuity, we structure this narrative accordingly:
  - **Abongomera et al. (2017), Kämink et al. (2017):** both models developed in East African MSF treatment centres (Ethiopia and South Sudan, respectively) and share a co-author.
  - **Costa et al. (2016)**, **Werneck et al. (2003)**: both models developed in the Instituto de Doenças Tropicais Nathan Portela (IDTNP), Teresina, state of Piauí, Brazil and share two co-authors.
  - **Coura-Vital et al. (2014), de Araújo et al. (2012)**: both models developed in Brazil using national registry data and share three co-authors.
  - **Sampaio et al. (2010), Foinquinos et al. (2021):** both models developed or updated in children <15 years, in IMIP (Instituto de Medicina Integral Professor Fernando Figueira), Recife, state of Pernambuco, Brazil, and share two co-authors.

## East African models

Both East African studies (Abongomera et al. and Kämink et al.) used retrospectively collected data from Médecins Sans Frontières (MSF) treatment centres and reported evaluations in datasets distinct from the development samples. The models were developed with an operational aim — to support patient management in MSF treatment settings.

### Abongomera et al. (2017) (3)

Abongomera et al. developed a prognostic model using patients who presented to a rural health centre in Abdurafi, Amhara region, Ethiopia (2008–2013). The development cohort comprised 1,686 patients, of whom 99 (5.9%) died in hospital. The model was externally evaluated in a neighbouring regional hospital (Leishmaniasis Research and Treatment Centre, Gondar) using 404 patients with 53 in-hospital deaths (13.1%) from 2011–2012. Both datasets were heavily male-dominated (>95%), had similar median ages (23–25 years), and relatively high HIV prevalence (19.3% in development; 13.6% in validation).

C-statistics were evaluated in the development and validation datasets: 0.83 (95% CI: 0.79-0.87) and 0.78 (95% CI: 0.72-0.83), respectively. Internal validation was performed using cross-validation in the development dataset, with an optimism-adjusted c-statistic of 0.82 (95% CI: 0.77-0.88).

Notably, Abongomera et al.’s model is the only East African model that included HIV as a predictor and is one of only two models overall to consider treatment as a candidate predictor (although treatment was not retained). It is also the sole model to both consider and retain tuberculosis co-infection.

The model was judged at high risk of bias for reasons common across the literature (see notes). The full regression equation is not published, so investigators wishing to perform an independent external validation would need to contact the authors.

### Kämink et al. (2017) (4)

Kämink et al. developed two prognostic models using patients presenting to Lankien hospital, Jonglei state, South Sudan (2013–2015): one for patients <19 years (n = 4,931; 116 in-hospital deaths, 2.4%) and one for patients ≥19 years (n = 1,702; 70 in-hospital deaths, 4.1%). Both derivation cohorts were ~55% male. Patients with known HIV/VL co-infection were excluded, limiting applicability to HIV-endemic settings.

The authors evaluated each model in three historical datasets: two from the same treatment centre (1999–2002 and 2002–2005) and one from a neighbouring MSF centre (Malakal hospital, Upper Nile state; 2002–2005).

In the derivation datasets the c-statistics were 0.83 (95% CI 0.78–0.87) for the <19 model and 0.74 (95% CI 0.68–0.81) for the ≥19 model. Across the six external evaluations (three per model), discrimination ranged from 0.71 to 0.83.

Reflecting the local disease presentation (South Sudan), this is the only model that considers lymphadenopathy as a candidate predictor, although not retained in the final model.

Both models were judged at high risk of bias for the common reasons listed above. The full model equations are not published; external validation would therefore require author contact.

## Brazilian models

### Costa et al. (2016) (5)

Of the six Brazilian studies, Costa et al. is the only study that both presents new model developments and subsequently evaluates them in new data (external validations). Four prognostic models are presented, developed from a prospective cohort of patients presenting to the Instituto de Doenças Tropicais Nathan Portela (IDTNP), Teresina, state of Piauí, between 2005 and 2008. Models developed by Costa et al. have seen widespread use in Brazil following their integration into the Brazilian national guidelines in 2011 (6,7).

Overall, 883 patients are described in the combined development datasets, of whom 553 (62.6%) were male, and 66 (7.5%) experienced in-hospital mortality. Where HIV was screened, 71/652 (10.9%) were positive. Two models were developed for patients ≥2 years: 569 patients using clinical characteristics alone and 538 patients using clinical and laboratory characteristics. Two further models were developed for patients <2 years: 314 patients using clinical characteristics alone, and 291 with clinical and laboratory characteristics. All four models were evaluated in two validation datasets, consisting of patients attending the same hospital: 449 patients between 2008 and 2009, and 582 patients between 2009 and 2013. Mortality events are not disaggregated by model-specific development or validation dataset.

Model discrimination (c-statistics) ranged from 0.89 to 0.93 in the development dataset, and from 0.71 to 0.92 in the validation datasets, with overall higher discrimination seen in the two <2 years models. Model calibration is assessed using the Hosmer-Lemeshow goodness-of-fit test, although this test has limited ability to evaluate poor calibration, and in the PROBAST risk of bias tool, is not considered a sufficient presentation of model calibration (1).

Both ≥2 years models considered and retained HIV as a mortality risk factor. Only one other Brazilian study, presenting one model, incorporates HIV as a predictor of mortality (Coura-Vital et al).

These are the only models with low risk of bias in the participant domain, given the study was prospective in nature. This allowed for consistent definitions and identification of predictor, participant and outcome information. A potential source of bias is the low number of mortality events compared to the high number of candidate predictors used, resulting in the events per predictor parameter (EPP) ratio being approximately 0.5 – 2.0 (where number of events are extrapolated to the different age groups based on the overall reported mortality rates). This raises concern for model overfitting and optimistic performance measures. Overfitting is likely partially responsible for the modest decrease in c-statistics when measured in the development vs validation datasets (apparent performance vs external validation).

Another important limitation is that the published risk scores cannot be reproduced from the reported regression coefficients and odds ratios (see Tables 2–4 of the original paper). For example, the <2-year clinical risk score includes “bleeding in 1–2 sites” and “dyspnoea”, yet associated coefficients are not provided; in the ≥2-year models the categorisation of bleeding sites differs between the risk score table and the coefficient table. While these discrepancies may reflect table preparation errors, they undermine confidence in score reproducibility (see Supplementary Text 3).

A further limitation, like many other models, is that the full model equations are not presented. The authors would need to be contacted by any researchers interested in evaluating the full models in new settings.

Costa et al. also used their development dataset (n = 883) to evaluate three further prognostic models, developed by Werneck et al, Coura-Vital et al, and Sampaio et al. (discussed below) (8–10). However, no information is provided on how the authors performed the external validation, with only c-statistics presented.

### Werneck et al. (2003) (8)

Werneck et al. published the earliest VL prognostic model in 2003 (8). Adopting a case-control design, 90 patients with VL were identified over an 18-month period, following review of hospital records in Teresina, state of Piauí (12 cases with mortality during treatment, 78 controls). The mean age was 14.2 years, and 68.9% were male. HIV status was not reported or included as a model predictor. Discrimination (c-statistic) was estimated at 0.88 (CI: not provided) in the development dataset.

When evaluated by Costa et al. in their Teresina hospital cohort, the c-statistic was estimated at 0.75 (CI: not provided) (5).

Of all identified models, Werneck et al. present the model with the fewest predictors (four predictors: duration of fever >60 days, haematocrit >20%, jaundice, diarrhoea), the smallest development dataset (90 patients), and second smallest number of outcome events (12 deaths). A significant risk of model overfitting is present due to a very low EPP (0.8). Owing to the case–control design, absolute mortality probabilities cannot be estimated, and the risk score is therefore not linked to mortality risk, serving only as a relative measure of mortality risk.

Given the case-control nature of the model, researchers would only be able to validate the model’s discrimination. Any intercept term, were it to be provided by the authors, would only reflect the authors’ choice of the case to control ratio.

### Coura-Vital et al. (2014) (10)

Coura-Vital et al. present a prognostic model using by far the largest development dataset of all models.

All patients with confirmed VL and without a missing outcome status were included from the Brazilian Notifiable Diseases Information System (SINAN: Sistema de Informação de Agravos de Notificação) between 2007 and 2011, with additional mortality data from the national Mortality Information System (SIM: Sistema de Informação sobre Mortalidade). Split-sample validation was performed: of the 18,501 eligible patients, two-thirds were selected at random (n = 12,333) for the development dataset, and one third (n = 6,168) for the validation dataset. Mortality due to VL was 770 (6.2%) and 386 (6.3%) in the development and validation datasets, respectively. Sex and HIV proportions were almost equal, with approximately 61% male and 7% with HIV co-infection. HIV was included as a candidate predictor and retained in the final model.

Discrimination (c-statistic), when measured in the development dataset was 0.80 (95% CI: 0.78-0.82), reducing to 0.78 (95% CI: 0.75-0.82) in the internal validation dataset, and to 0.77 (CI: not provided) when evaluated in the Teresina cohort by Costa et al. (5).

Coura-Vital’s study presents the only model development that does not suffer from overfitting due to a small sample size, with an EPP of 26.55 in the development dataset, and 386 mortality events in the validation dataset. Furthermore, the full model equation is presented, so authors would not need to be contacted if model validation were to be performed in a new setting. This is also the only model where predicted and observed probability estimates are presented (Tables 3 and 4 of the publication), allowing for the construction of a calibration plot if desired.

Several limitations arise from reliance on registry data. The timing of death relative to presentation is not always clear, and predictor information in surveillance systems can suffer from inconsistent definitions, variable completeness, and misclassification.

### de Araújo et al. (2014) (11)

de Araújo et al. developed a prognostic model in patients reported to the Brazilian Notifiable Diseases Information System (SINAN) between 2007 and 2009, and who were also resident in Belo Horizonte, with suspected or confirmed VL. Similar to Coura-Vital et al, mortality data was supplemented from the national Mortality Information System (SIM). A total of 376 patients were identified, of whom mortality due to VL occurred in 49 (13.0%).

Both de Araújo et al. and Coura-Vital et al. drew from the same national registry and therefore considered many of the same candidate predictors. Nevertheless, the final predictor sets differ (Figure 2), reflecting differences in the development cohorts: de Araújo et al. used a geographical and temporal subset of the registry (residents of Belo Horizonte, 2007–2009) and included both suspected and confirmed cases, whereas Coura-Vital restricted their analysis to confirmed cases

Similar sources of bias exist as seen with other models, including univariable selection, the use of registry data, and a particularly low EPP of 1.02, raising concerns for model overfitting. Furthermore, the presented risk score is not linked to estimate mortality probabilities, so only relative mortality risk can be inferred.

Researchers wanting to validate their model in new data would need to contact the authors for the full model equation.

### Sampaio et al. (2010) (9)

Sampaio et al. developed a model using a retrospective hospital cohort of patients <15 years who attended a hospital in Recife (Instituto de Medicina Integral Prof. Fernando Figueira), state of Pernambuco, between 1996 and 2006. A total of 546 patients were identified, with 57 (10.4%) experiencing in-hospital mortality. The median age was 3.2 years, and approximately half the patients were male.

The model was found to have a c-statistic 0.895 (CI: not provided) when evaluated in the development dataset. Two external validations of the model were performed: (i) Costa et al. estimated a c-statistic of 0.87 (CI: not provided) when measured in their Teresina cohort (5), and (ii) Foinquinos et al. estimated a lower c-statistic of 0.62 (CI: not provided) when evaluating the *full* model in their dataset, leading to model updating (logistic recalibration, see below) (12).

Sampaio et al.’s model is subject to all the common sources of bias described in the notes. Researchers wishing to externally validate their model can find the full model equation presented by Foinquinos et al.(12).

### Foinquinos et al. (2021) (12)

Foinquinos et al. evaluated and updated the model developed by Sampaio et al. using a dataset from the same hospital in Recife.

The authors included 156 patients, also <15 years, identified from hospital records and attending IMIP between 2008 and 2018 (the updating dataset). 10 patients (6.4%) experienced in-hospital mortality. When validating Sampaio et al.’s model, the c-statistic was estimated at 0.62 (CI: not provided). A Hosmer-Lemeshow goodness-of-fit test and Spiegelhalter test both showed evidence of poor calibration.

The authors applied logistic recalibration (adjusting the calibration slope and intercept) and, on re-evaluation, the model’s discrimination increased to a c-statistic of 0.71 (95% CI 0.62–0.90). Unlike many studies that report discrimination for simplified risk scores, the c-statistics reported here appear to reflect the performance of the full (recalibrated) regression model rather than a points-based score. Given the updating dataset included only 10 mortality events, however, these estimates are imprecise.

Given this study only performs validation and updating, there were no candidate predictors, and the final model predictors remain unchanged from Sampaio et al.’s model.

With only 10 mortality events in the updating dataset, this represents the dataset with the lowest number of outcome events, leading to significant uncertainty in the estimated c-statistics.

The full model equation is available for researchers wishing to evaluate the model in new settings.

## References

1. Moons KGM, Wolff RF, Riley RD, Whiting PF, Westwood M, Collins GS, et al. PROBAST: A Tool to Assess Risk of Bias and Applicability of Prediction Model Studies: Explanation and Elaboration. Ann Intern Med. 2019 Jan 1;170(1):W1.

2. White N, Parsons R, Collins G, Barnett A. Evidence of questionable research practices in clinical prediction models. BMC Med. 2023 Sept 4;21(1):339.

3. Abongomera C, Ritmeijer K, Vogt F, Buyze J, Mekonnen Z, Admassu H, et al. Development and external validation of a clinical prognostic score for death in visceral leishmaniasis patients in a high HIV co-infection burden area in Ethiopia. Satoskar AR, editor. PLoS ONE. 2017 June 5;12(6):e0178996.

4. Kämink SS, Collin SM, Harrison T, Gatluak F, Mullahzada AW, Ritmeijer K. A clinical severity scoring system for visceral leishmaniasis in immunocompetent patients in South Sudan. Lockwood DNJ, editor. PLoS Negl Trop Dis. 2017 Oct 2;11(10):e0005921.

5. Costa DL, Rocha RL, Chaves EDBF, Batista VGDV, Costa HL, Costa CHN. Predicting death from kala-azar: construction, development, and validation of a score set and accompanying software. Rev Soc Bras Med Trop. 2016 Dec;49(6):728–40.

6. Ministry of Health (Brazil). Visceral leishmaniasis: clinical recommendations for lethality reduction (Leishmaniose visceral : recomendações clínicas para redução da letalidade) [Internet]. 1st edn. Brasilia, Brazil; 2011. Available from: https://bvsms.saude.gov.br/bvs/publicacoes/leishmaniose_visceral_reducao_letalidade.pdf

7. Ministry of Health (Brazil). Guide to Health Surveillance: volume 2. 6th Edition. (Guia de Vigilância em Saúde. 6^a^ edição revisada) [Internet]. 6th edn. Brasilia, Brazil; 2024 [cited 2025 May 29]. 1011 p. Available from: https://bvsms.saude.gov.br/bvs/publicacoes/guia_vigilancia_saude_v2_6edrev.pdf

8. Werneck GL, Batista MSA, Gomes JRB, Costa DL, Costa CHN. Prognostic Factors for Death from Visceral Leishmaniasis in Teresina, Brazil. Infection. 2003 June;31(3):174–7.

9. Sampaio MJADQ, Cavalcanti NV, Alves JGB, Fernandes Filho MJC, Correia JB. Risk Factors for Death in Children with Visceral Leishmaniasis. Franco-Paredes C, editor. PLoS Negl Trop Dis. 2010 Nov 2;4(11):e877.

10. Coura-Vital W, Araújo VEMD, Reis IA, Amancio FF, Reis AB, Carneiro M. Prognostic Factors and Scoring System for Death from Visceral Leishmaniasis: An Historical Cohort Study in Brazil. Santiago HDC, editor. PLoS Negl Trop Dis. 2014 Dec 11;8(12):e3374.

11. De Araújo VEM, Morais MHF, Reis IA, Rabello A, Carneiro M. Early Clinical Manifestations Associated with Death from Visceral Leishmaniasis. Reithinger R, editor. PLoS Negl Trop Dis. 2012 Feb 7;6(2):e1511.

12. Foinquinos J, Duarte MDC, Figueiroa JN, Correia JB, Cavalcanti NV. Temporal Validation of a Predictive Score for Death in Children with Visceral Leishmaniasis. Wang M, editor. Journal of Tropical Medicine. 2021 Dec 22;2021:1–6.
