# Supplementary material for: Prognostic models predicting clinical outcomes in patients diagnosed with visceral leishmaniasis: a systematic review
Source: BMJ Public Health. 2026 Mar 27;4(1):e001196. doi: 10.1136/bmjph-2024-001196 (PMC13034366; doi:10.1136/bmjph-2024-001196)
Supplement: online supplemental file 3 [file bmjph-4-1-s009.docx]

# This is a R script written in base R (R version 4.3.1).

# Date: August 2023.

# Author: James Wilson.

# For each study and model development, we use the presented regression coefficients

# (log odds ratios) to derive the simplified score weights according to the study’s

# methodology

# where the regression coefficients do not correspond to the score weights, differences

# introduced from rounding errors are considered (for example, due to the number of decimal

# places reported in the published tables of regression coefficients)

# many studies reference the method used by Barquet et al for the derivation of risk scores from

# the logistic regression coefficients

## "Points were assigned to each prognostic factor by dividing the coefficient of each predictor from

## the logistic regression analysis by the smallest coefficient, then rounding each quotient to the nearest

## integer. Finally, a prediction score was calculated for each patient by summing the total number of points."

# Barquet N, et al. Prognostic factors in meningococcal disease. Development of a bedside

# predictive model and scoring system. Barcelona Meningococcal Disease Surveillance Group. JAMA.

# 1997 Aug 13;278(6):491-6. doi: 10.1001/jama.278.6.491. PMID: 9256224.

# rc: regression coefficients (log odds ratios)

# aoc: adjusted odds ratios (where regression coefficients not presented)

# rc = natural log (aoc) = ln(aoc)

######################

### de Araujo 2012 ###

######################

rc <- c(

"other infections" = 1.17,

"bleeding" = 1.25,

"jaundice" = 2.31,

"age_60" = 1.13

)

round(rc / min(rc)) # score is reproducible

# Methodology: Barquet et al. referenced

########################

### Coura-Vital 2014 ###

########################

rc <- c(

"splenomegaly" = 0.43,

"edema" = 0.57,

"weakness" = 0.50,

"bleeding" = 1.34,

"jaundice" = 0.48,

"age_0.5" = 2.14,

"age_0.5_1" = 1.02,

"age_19_50" = 0.87,

"age_50_65" = 1.34,

"age_65" = 2.25,

"leishmania-HIV co-infection" = 0.50,

"bacterial infection" = 0.63

)

round(rc / min(rc)) # score is reproducible

# methodology: Barquet et al. referenced

####################

### Werneck 2003 ###

####################

aor <- c(

"diarrhea" = 7.82,

"jaundice" = 10.6,

"fever" = 8.64,

"hematocrit" = 15.9

)

rc <- log(aor)

round(rc / min(rc)) # score is reproducible

# methodology: Barquet et al. referenced

####################

### Sampaio 2010 ###

####################

aor <- c(

"jaundice" = 4.4,

"dyspnoea" = 2.8,

"mucosal bleeding" = 4.1,

"associated infections" = 2.7,

"platelets" = 11.7,

"neutrophils" = 3.1

)

rc <- log(aor)

round(rc / min(rc)) # not reproducible following Barquet's methodology

round(rc / 0.8) # now it's reproducible - so risk scores are consistent with regression coefficients, but using different scaling

# methodology: Barquet et al. referenced

#########

# correlation with coefficients from Foinquinos 2021

rc <- c(

"jaundice" = 1.490908,

"dyspnoea" = 1.015233,

"mucosal bleeding" = 1.415629,

"associated infections" = 1.003439, # denominator

"platelets" = 2.458888,

"neutrophils" = 1.145403

)

exp(rc) # appear consistent

round(rc / min(rc)) # not reproducible

round(rc / 0.8)

#######################

### Foinquinos 2021 ###

#######################

rc <- c(

"jaundice" = 1.490908,

"dyspnea" = 1.015233,

"bleeding" = 1.415629,

"infections" = 1.003439,

"platelets" = 2.458888,

"neutrophils" = 1.145403

)

rc_update <- rc * 0.5224832

round((rc_update) / min(rc_update)) # not reproducible

# methodology: Barquet et al (referenced by Sampaio et al)

##################

### Costa 2016 ###

##################

# Regression coefficients reported in Tables 2 and 3

# Risk score coefficients reported in Table 4

# model: < 2 years, clin.

rc <- c(

"age < 12" = 1.4, # groups switched in article

"bleeding 3-4 sites" = 1.8,

"bleeding 5-6 sites" = 3.6,

"edema" = 1.8,

"jaundice" = 0.9

)

# included in the risk score, but not presented as regression coefficients: bleeding 1-2 sites, and dysnea coefficients

rc / min(rc) # not reproducible (score includes dyspnoea)

# model: < 2 years, clin./lab.

rc <- c(

"age < 12" = 1.1,

"bleeding 3-4 sites" = 1.8,

"bleeding 5-6 sites" = 4.1,

"edema" = 1.4,

"dyspnea" = 1.1,

"AST/ALT" = 2.5

)

# included in the risk score, but not presented as regression coefficients: bleeding 1-2 sites

rc / min(rc) # not reproducible

rc / 0.95 # changing the denominator does not reproduce scores

2.55 / 1.05 # max possible score for AST/ALT with rounding error

# model: > or equal to 2 years, clin.

aor <- c(

"age >40" = 3.5,

"2 sites(?exp)" = 2.3, # inconsistent with rc, exp(2.3)

"3 or more sites" = 10.4, # inconsistent with rc, exp(2.6)

"HIV" = 6.1,

"edema" = 2.2,

"jaundice" = 2.9,

"dyspnea" = 2.3,

"vomiting" = 2.0,

"bac infection" = 2.9

)

rc <- c(

"age >40" = 1.3,

"2 sites(?exp)" = 2.3, # inconsistent with rc, log(2.3)

"3 or more sites" = 2.6, # inconsistent with rc, log(10.4)

"HIV" = 1.8,

"edema" = 0.8,

"jaundice" = 1.1,

"dyspnea" = 0.8,

"vomiting" = 0.7,

"bac infection" = 1.1

)

# included in the risk score, but not presented as regression coefficients: age 15-40 years, bleeding categories differ

log(aor)

log(aor) / min(log(aor))

round(log(aor) / min(log(aor))) # not reproducible (different grouping)

# model: > or equal to 2 years, clin./lab.

rc <- c(

"3 or more sites" = 1.7, # not significant predictor

"hiv" = 2.2,

"jaundice" = 1.0,

"dyspnea" = 0.8,

"bac infection" = 1.2,

"leuk" = 1.3,

"plat" = 2.0,

"renal fail" = 2.4

)

# "3 or more sites" does not appear in risk score

rc / min(rc)

round(rc / min(rc)) # not reproducible

# methodology: Barquet et al. referenced

#######################

### Abongomera 2017 ###

#######################

alhr <- c(

">40" = 2.22,

"5-18" = 0.93,

"HIV pos" = 3.04,

"HIV neg" = 0.54,

"Hb < 6.5" = 2.16,

"Hb > 6.5" = 0.67,

"Bleeding +" = 3.11,

"Bleeding -" = 0.93,

"Jaundice +" = 3.21,

"Jaundice -" = 0.93,

"Weakness +" = 1.64,

"Weakness -" = 0.84,

"Edema -" = 0.84,

"Edema +" = 2.36,

"Ascites +" = 5.84,

"Ascites -" = 0.92,

"TB +" = 1.71,

"TB -" = 0.92

)

alhr_adj <- alhr[alhr >= 1.5 | alhr < 0.67]

round(log(alhr_adj)) # reproducible

# methodology: Speigalhalter-Knill-Jones (Barquet not referenced)

###################

### Kamink 2017 ###

###################

rc <- c(

"age_2_5" = 0.98,

"age_2" = 2.37,

"weak_sev" = 0.92,

"weak_col" = 3.13,

"hb_75_89" = 0.57,

"hb_60_74" = 1.02,

"hb_60" = 2.05,

"jaundice" = 2.55

)

round(rc / min(rc)) # reproducible

rc <- c(

"weak_sev" = 0.90,

"weak_col" = 1.44,

"bmi_145_159" = 0.96,

"bmi_130_144" = 1.02,

"bmi_13" = 1.58,

# "hb_75_89" = 0.07,

# "hb_60_74". = 0.42,

"hb_60" = 1.50,

"oed_asc" = 1.58,

"jaundice" = 1.23

)

round(rc / min(rc)) # reproducible

# methodology: Barquet et al. referenced
